# Editorial: Eye Pain: Etiology and Therapeutic Approaches

**DOI:** 10.3389/fphar.2022.914809

**Published:** 2022-04-27

**Authors:** Dario Rusciano, Paola Bagnoli, Juana Gallar, Anat Galor

**Affiliations:** ^1^ Fidia Research Center, Catania, Italy; ^2^ University of Pisa, Pisa, Italy; ^3^ Instituto de Neurociencias, Universidad Miguel Hernandez-CSIC, San Juan de Alicante, Spain; ^4^ Bascom Palmer Eye Institute, University of Miami School of Medicine, Miami, FL, United States

**Keywords:** eye pain, neuropathic pain, nociceptive pain, dry eye, cornea

The eye is heavily innervated by sensory nerve fibers. Corneal sensory nerves sprout from the ophthalmic division of the trigeminal nerve, travel in the nasociliary nerve and its branches, finally entering the cornea through the sclera and the conjunctiva. They form a stromal network supplying different regions of the cornea: midstromal, sub-basal/subepithelial and epithelial. The receptive fields of corneal sensory receptors are large and partially overlapping, thus resulting in poor localization or acuity, but producing a very high level of sensitivity to external stimuli. The central corneal nerve density is approximately 7,000 nerve endings per mm^2^, so that cornea sensitivity is 300–600 times higher than skin, and 20–40 times greater than dental pulp. Different types of corneal sensory nerves have been characterized. Approximately 20% belong to the class of mechano-nociceptive receptors responding to mechanical stimuli and responsible for acute sharp pain conducted through thin myelinated fibers. Some 70% are polymodal nociceptors responding to chemical mediators, heat and irritants through slow-conducting, unmyelinated nerve fibers. Finally, 10% are cold receptor fibers activated by cold solutions or cold air, such as it may happen during tear film evaporation. Beside these relevant sensory functions, corneal nerves also regulate reflex tear production and the associated blinking reflex, and contribute to the release of trophic factors, such as substance-P, NGF, KGF, CNTF, PDGF-B, TGF-α and IL1β. In fact, iatrogenic or traumatic damage to corneal sensory fibers may result in neurotrophic keratopathy, characterized by epithelial cell loss and edema. Being so sensitive, the cornea is susceptible to pain. Pain protects tissue from injury. Painful stimuli detected by nociceptors are transmitted *via* action potentials to higher order centers where the pain is perceived.

Pain can be acute, when of high intensity and lasting a short time, or chronic, when its duration reaches and extends over 3 months. Depending on the stimulus triggering pain, it can be differentiated in nociceptive (caused by the physiological response to a noxious event) or neuropathic, when the algic response results from a dysfunction caused by damage of the sensory system (either the peripheral sensory nerves or the central neurons) and hardly treated by topical analgesics when central mechanisms are involved. Chronic pain may have a neuropathic component. Perturbations of the eye surface such as dry-eye, pterygia or conjunctivochalasis, inflammation and infections may be triggers of eye pain. This kind of pain is typically treated by topical antinflammatory agents and ointments, or anesthetics. When eye pain is reported out of proportion to clinical signs, or with no apparent previous insult, neuropathic pain is suspected.

Neuropathic pain is not a reaction to noxious stimuli, rather it is the result from an insult to the nervous system. During regeneration of damaged corneal nerves there is an increase in the expression of ion channels involved in their excitability, which may produce spontaneous activity and a low activation threshold. This altered activity may influence the synaptic connections along the sensory pathway, leading to permanent changes at central levels. To complicate matters, the severity of eye pain, that can be unilateral or bilateral, does not indicate how serious the underlying cause of the discomfort is. Symptoms of neuropathic corneal pain may include sensitivity to light and air, foreign body sensation, burning and severe eye dryness. In fact, symptoms of neuropathic corneal pain may sometimes be confused with dry eye syndrome, although the signs typical of this disease are missing. In fact, patients with neuropathic corneal pain do not respond to usual dry eye treatments. Neuropathic corneal pain can result from eye surgery, chronic dry eye disease generated or aggravated by the use of preservatives in eye drops; extended use of contact lenses; diabetes; neuralgia of the trigeminus. It can be associated with anxiety or depression, headache or migraine, fibromyalgia and autoimmune diseases.

Severe pain sensation and light sensitivity prevent those afflicted with ocular neuropathic pain from performing activities of daily living and is associated with symptoms of anxiety and depression.

While nociceptive pain is targeted through the use of topical therapies, neuropathic pain can be treated with oral agents or adjunctive therapies if a neuropathic component is highly suspected. While many studies have examined treatment outcomes for nociceptive sources of ocular pain, fewer have examined outcomes after treatment of neuropathic ocular pain. Strategies to counteract neuropathic ocular pain include ocular surface treatment, anti-inflammatory compounds, serum containing growth factors which play a crucial role in neuroregeneration and healing, anticonvulsants, opioid agonists and alternative therapies.

To date, however, comprehensive understanding of the mechanisms underlying ocular neuropathic pain is still under exploration and efficient treatment of neuropathic eye pain has yet to be found. The challenge is a better definition of the molecular targets of neuropathic eye pain, and the identification of specific therapeutic agents to be given either as topical or as systemic treatment.

Therefore, given the relatively high frequency of eye pain, the multiplicity of its causes (at least 22 possible causes have been described), and the complexity of neuropathic pain, the aim of this Research Topic has been to collect a series of recent studies focusing on the different aspects of ocular pain, its molecular triggers and innovative treatment strategies.

## Pain Mechanisms and Perception

The thresholds for subjective perception of corneal sensing receptors to different stimuli (cold, mechanical and chemical) applied at increasing intensities is addressed in the manuscript presented by Jayakumar and Simpson, in order to try and dissect patient’s processing of the stimulus in the two phases of detection and response.

Among the several stimuli that are known to activate peripheral terminals of trigeminal sensory neurons at the cornea, conjunctiva and sclera, acidic stimuli have been shown to induce the firing of polymodal nociceptors through the activation of specific ion channels. In the paper of Comes et al., ion channels and receptors that are involved in acid sensing are reviewed. Because of the acid environment in the cornea and the conjunctiva, a number of compounds used to treat eye diseases are formulated in acidic solutions to facilitate their solubilization and absorption through the cornea. Despite some of the mechanisms underlying proton sensing in the ocular surface have been elucidated, further studies are needed to clarify the differential role of channels or membrane receptors which might allow to develop specific therapeutic interventions.

A review of Puja et al. describes the recent advances on the role of molecular and cellular mechanisms contributing to peripheral and central pain sensitization of the trigeminal pathway, together with mechanisms underlying corneal sensory transduction and peripheral pain sensitization in the trigeminal spinal nucleus.

The brain networks related to pain processing have been extensively studied with functional neuroimaging over the past 20 years. Based on these observations, supraspinal mechanisms underlying ocular pain are detailed by Pondelis and Moulton, describing the anatomy and the physiology of the different brain regions that receive afferent inputs from the trigeminal system. In the case of nociception, nociceptors’ signals traveling through supraspinal centers finally reach the cortex where the pain sensation is generated. On the other hand, neuropathic pain is generated by alterations in the somatosensory nervous system, not necessarily involving peripheral receptors. Clarifying the neural pathways at the origin of neuropathic ocular pain is critical to understanding its mechanisms and ultimately its treatment.

Dry eye disease is often associated with neuropathic ocular pain. Although it is mostly generated by nociceptive stimulation induced by alterations of tear film dynamics, chronic dryness lead to nerve damage and induce morpho-functional changes of corneal nerves. In this context, persistent ocular pain in the absence of detectable signs can be considered a form of neuropathic pain. In the paper by Bereiter et al., the authors try to clarify the basis of ocular hyperalgesia in animal models of dry eye disease by demonstrating that the activation of P2X7R, a purinergic receptor expressed by non-neural cells in the trigeminal nerve pathway, contributes to ocular hyperalgesia and to microglia activation in both male and female animals, an effect that is further amplified by estrogen treatment in females.

Finally, in a preclinical study, Luna et al. using the guinea pig model, provided the first demonstration that a unilateral lesion of the corneal nerves affects the corneal sensitivity in both the ipsilateral and the contralateral eye. This is in line with the clinical finding that some patients with unilateral ocular alteration reported discomfort and pain also in the contralateral eye. Although the mechanisms underlying the contralateral alteration of sensory nerves remains to be determined, available data support the involvement of neuroimmune interactions. These findings imply that in preclinical and clinical studies the contralateral eye cannot be used as a control and that in clinical practice both eyes need to be treated also in the presence of unilateral ocular damage.

## Ocular Pain Handling

In ocular pain handling, preoperative management has been focused to the use of musicotherapy in patients undergoing cataract surgery, the most frequently performed surgical procedure. In a paper by Guerrier et al., a prospective controlled trial including 243 patients has shown that music intervention prior to the surgery can reduce anxiety level and self-reported pain intensity both during cataract surgery under local anesthesia and in the early postoperative period. The underlying mechanisms remain unclear, although molecular mechanisms related to opioid and cytokine metabolism are discussed together with psychophysiological mechanisms bringing to anxiety reduction.

In an opinion article, Santarcangelo and Carli, two experts in pain management, discuss the effectiveness of psychological interventions focused mainly on hypnosis for disease management. In particular, hypnotizability is used as a model to support the view that specific psycho-physiological traits and cognitive strategies can not only reduce pain, but also modulate the pain-related autonomic and immune activity, induce cortical plasticity relevant to pain control, and assist in the choice of the most appropriated treatment.

Topical treatments have been dealt with in four different articles. In a pilot study, Delicado-Miralles et al. investigate the effects of F6H8, an alkane previously shown to alleviate dry-eye associated symptoms, on a healthy ocular surface. Through corneal surface temperature regulation, F6H8 has been shown to increase blinking and tearing thus contributing to alleviate dry eye disease and additional ocular pathologies.

Major efforts are aimed at developing topical therapeutic options to treat neuropathic pain of the cornea. After providing the criteria to distinguish patients with corneal neuropathic pain from those with non-neuropathic ocular discomfort that can be associated with inflammation or dry eye disease, Nortey et al. revise the findings on the efficacy of topical corticosteroids in patients with dry eye and corneal neuropathic pain. In corneal neuropathic pain, serum tears have been described to be of some help in patients experiencing discomfort to light. In addition, topical lacosamide has been shown to exert beneficial effects by decreasing the hyperexcitability of corneal cold-sensitive nerve terminals. Finally, eye drops of naltrexone, an opioid antagonist, have been found to ameliorate corneal neuropathic problems and their efficacy are under active investigation together with topical encephalin modulators as potential pain therapeutics.

Nociceptive pain is targeted through the use of topical therapies, and oral agents or adjunctive therapies can be used if a neuropathic component is highly suspected. Treatment outcomes for nociceptive ocular pain have been more studied than those for neuropathic ocular pain, mostly because most therapies are oriented against nociceptive inflammatory ocular pain, and less have been focused against neuropathic ocular pain. In a retrospective study involving patients with a clinically diagnosed neuropathic ocular surface pain, Patel et al. examine the individual response to different treatments with the aim of studying subjective clinical responses to a number of commonly utilized medications. The individual variability in treatment responses points to the necessity of future research aimed to develop diagnostic tests that can localize nervous system abnormalities together with application of personalized approaches that combine oral, topical or adjuvant medications.

Preclinical evidence about the efficacy of topical gabapentin on neuropathic ocular pain is provided by Cammalleri et al. in a rabbit model system in which eye drops with gabapentin exert analgesic effects coupled to stimulation of tear secretion. Secretagogue efficacy of gabapentin involves both a stimulation of the autonomic nervous system and a direct activation of intracellular signaling cascades, including the PKA/CREB pathway, culminating in the increased expression of aquaporin 5 in the lacrimal gland through mechanisms that remain to be elucidated.

In conclusion, we believe that the collection of papers that are included in this Research Topic represent the state of the art of the present knowledge on corneal pain, and we hope that it can be of inspiration to those scientists who are working on this subject, and to those who are approaching this fascinating research topic.

